# Zanubrutinib Combined With Venetoclax for the Treatment of Central Nervous System‐Involved Chronic Lymphocytic Leukemia/Small Lymphocytic Lymphoma: A Rare Case Report

**DOI:** 10.1002/cnr2.70733

**Published:** 2026-11-10

**Authors:** Wei Xie, Yanxue Yin, Jing Wang, Guohui Cui, Lu Zhang

**Affiliations:** ^1^ Institute of Hematology, Union Hospital, Tongji Medical College, Huazhong University of Science and Technology Wuhan China; ^2^ Department of Radiology, Union Hospital, Tongji Medical College Huazhong University of Science and Technology Wuhan China

**Keywords:** case report, central nervous system (CNS), chronic lymphocytic leukemia/small lymphocytic lymphoma (CLL/SLL), venetoclax, zanubrutinib

## Abstract

**Background:**

Clinically significant CNS involvement by CLL/SLL is uncommon, diagnostically challenging, and has no established standard treatment.

**Case:**

A 61‐year‐old man with previously untreated CLL/SLL was admitted in April 2023 to Union Hospital, Tongji Medical College, Huazhong University of Science and Technology, Wuhan, China, with rapidly progressive paraparesis, sensory disturbance, urinary retention, B symptoms, and bulky lymphadenopathy. Baseline white blood cell count was 2.30 × 10^9^/L, absolute lymphocyte count 1.48 × 10^9^/L, hemoglobin 82 g/L, platelet count 157 × 10^9^/L, and β2‐microglobulin 8.3 mg/L. CSF flow cytometry identified 46.31% abnormal monoclonal mature small B cells, and IgH/IgK rearrangements were detected. Whole‐spine magnetic resonance imaging showed abnormalities involving the lower medulla oblongata and spinal cord. After dexamethasone and intrathecal chemotherapy, zanubrutinib 160 mg twice daily was initiated in April 2023; venetoclax was started later that month at 20 mg daily and escalated weekly to 400 mg daily. Independent ambulation was restored by Month 3. At Month 4, marrow and CSF cytology/flow cytometry were negative and the largest abdominal mass had decreased from 11.0 × 6.4 to 1.3 × 0.9 cm. Low‐level CSF measurable residual disease (MRD), assessed by flow cytometry through detection of 57 phenotypically abnormal monoclonal mature small B cells (2.22% of nucleated cells), was detected in June 2024 and became negative after lumbar puncture and intrathecal therapy in November 2024. PET/CT in December 2024 showed a Deauville score of 2. Venetoclax was discontinued in April 2025 after approximately 2 years of combination therapy, and zanubrutinib monotherapy was continued. At final follow‐up in April 2026, the patient remained clinically stable with normal bowel and bladder function; marrow and CSF examinations were negative, and brain and whole‐spine MRI showed no intracranial or intraspinal tumor involvement. Blood counts remained generally stable, and no clinically significant toxicity was documented.

**Conclusion:**

This case supports further investigation of zanubrutinib plus venetoclax as the systemic component of a multimodal treatment strategy for CNS‐involved CLL/SLL. Because corticosteroid and intrathecal therapy were administered concomitantly, and because this is a single case, the independent efficacy and general safety of the oral combination cannot be established.

## 1 Introduction

Clinically significant CNS involvement by CLL/SLL is rare. In the largest single‐center cohort, clinically significant CNS involvement was diagnosed in 0.4% of the overall CLL/SLL population, and neurologic symptoms were more frequently caused by infection, autoimmune or inflammatory disease, Richter transformation, or unrelated conditions than by CLL/SLL itself [1]. Contemporary reviews continue to emphasize the absence of a standardized diagnostic or therapeutic approach [2, 3].

Targeted therapy is biologically attractive in this setting. Ibrutinib‐ and venetoclax‐based approaches have produced responses in individual patients with CNS involvement by CLL/SLL [4, 5, 6, 7, 8], and zanubrutinib has demonstrated measurable CSF exposure in a CNS lymphoma series [9]. BTK inhibition can increase mitochondrial dependence on BCL‐2 and enhance venetoclax sensitivity [10]. Zanubrutinib is an established systemic therapy for CLL/SLL [11, 12], and zanubrutinib‐containing venetoclax combinations have produced deep responses in systemic CLL/SLL studies [13, 14]. Class‐level evidence from fixed‐duration BTK inhibitor–venetoclax trials further supports the biological and clinical rationale for dual targeting [15, 16].

The present case is unusual because it combined severe spinal cord and lower medullary involvement, a high CSF clonal B‐cell burden, major neurologic disability, bulky systemic CLL/SLL, and long‐term serial CSF and imaging follow‐up. To our knowledge, the published literature has not previously described this exact zanubrutinib–venetoclax doublet in CNS‐involved CLL/SLL. Nevertheless, dexamethasone and intrathecal chemotherapy were given before and during systemic treatment, so causality cannot be assigned to the oral agents alone.

## 2 Case Presentation

A 61‐year‐old man was admitted in April 2023 to Union Hospital, Tongji Medical College, Huazhong University of Science and Technology, Wuhan, China. He had first undergone PET/CT in March 2022 because of lymphadenopathy present for approximately 3 years. Right axillary lymph‐node biopsy in April 2022 established CLL/SLL. The tumor cells expressed CD20, CD5, CD23, LEF1, CD200, BCL2, and CD21 and were negative for CD3, CD10, BCL6, CD30, CD15, IgD, cyclin D1, SOX11, and EBER; MUM1 was partially positive, p53 was weakly positive in approximately 20% of cells, and the Ki‐67 labeling index was approximately 5%. Molecular testing detected an IGH(D–J) rearrangement and an unmutated/wild‐type IGHV status involving the IGHV3‐30 family. Because he initially lacked a treatment indication, he was observed without therapy.

Intermittent night sweats and fatigue developed in October 2022. During the 3 months before the April 2023 admission, these symptoms worsened and were accompanied by a 15‐kg weight loss. During the month before admission, progressive bilateral lower‐limb weakness, numbness involving the limbs and abdominal skin, constipation, lower abdominal distension, and incomplete urination developed. He was wheelchair‐bound on admission. ECOG performance status was 4.

Physical examination showed bilateral submandibular and axillary lymphadenopathy; the largest left axillary node was approximately 5 × 5 cm. A palpable abdominal mass measured approximately 12 × 10 cm. Left and right lower‐limb muscle strength were Grade 3/5 and Grade 4/5, respectively. Pain sensation was reduced in the fingers, feet, and abdominal skin. Baseline laboratory studies showed a white blood cell count of 2.30 × 10^9^/L, absolute lymphocyte count of 1.48 × 10^9^/L, hemoglobin of 82 g/L, platelet count of 157 × 10^9^/L, β2‐microglobulin of 8.3 mg/L, and high‐sensitivity C‐reactive protein of 0.81 mg/L. Free triiodothyronine was 2.1 pmol/L, with free thyroxine of 14.0 pmol/L and thyroid‐stimulating hormone of 2.2850 μIU/mL. Direct antiglobulin tests for IgG and C3 and the indirect antiglobulin test were negative. Plasma CMV DNA was below the quantification limit (< 4.00 × 10^2^ copies/mL), whereas plasma EBV DNA was 2.66 × 10^3^ copies/mL; cell‐associated whole‐blood CMV and EBV DNA levels were 4.22 × 10^2^ and 2.04 × 10^6^ copies/mL, respectively.

Bone‐marrow biopsy showed small‐cell atypical infiltration involving approximately 20% of marrow cellularity. The atypical cells were positive for CD20 (partial), PAX5, CD5 (weak), CD23, LEF1 (partial/weak), and CD21 and negative for CD3, cyclin D1, SOX11, CD30, and EBER. Bone‐marrow cytology showed lymphocytes accounting for 61% of nucleated cells. Flow cytometry identified 24.21% abnormal monoclonal mature small B cells, with partial CD5 positivity, CD10 negativity, and a CLL score of 5. Conventional karyotyping and fluorescence in situ hybridization were reported as normal.

CSF was clear and colorless, with a positive Pandy test. The total cell count and leukocyte count were each 316 × 10^6^/L; mononuclear and polymorphonuclear cells accounted for 99% and 1%, respectively. CSF chemistry showed protein 0.87 g/L, glucose 3.68 mmol/L, chloride 117.1 mmol/L, adenosine deaminase 1 U/L, aspartate aminotransferase 16 U/L, lactate dehydrogenase 29 U/L, and creatine kinase 2 U/L. CSF cytology showed a small number of suspicious tumor cells. CSF flow cytometry identified 46.31% abnormal monoclonal mature small B cells, and IgH and IgK gene rearrangements were detected. These findings, together with the neurologic syndrome and imaging abnormalities, supported CNS involvement by CLL/SLL.

Contrast‐enhanced CT showed widespread cervical, supraclavicular, axillary, mediastinal, abdominal‐pelvic, inguinal, and cardiophrenic lymphadenopathy, with the largest lesion measuring approximately 11.0 × 6.4 cm. Nasopharyngeal soft‐tissue thickening was present. PET/CT in April 2023 showed progressive hypermetabolic lymphadenopathy, newly enlarged and hypermetabolic spleen, diffuse increased spinal‐cord metabolism, urinary retention consistent with neurogenic bladder, and hypermetabolic thickening of the posterior nasopharyngeal wall. Whole‐spine MRI in April 2023 showed abnormal signals in the lower medulla oblongata and spinal cord and a suspicious pontine signal (Figure 1A(a),B(a),C(a)).

**FIGURE 1:**
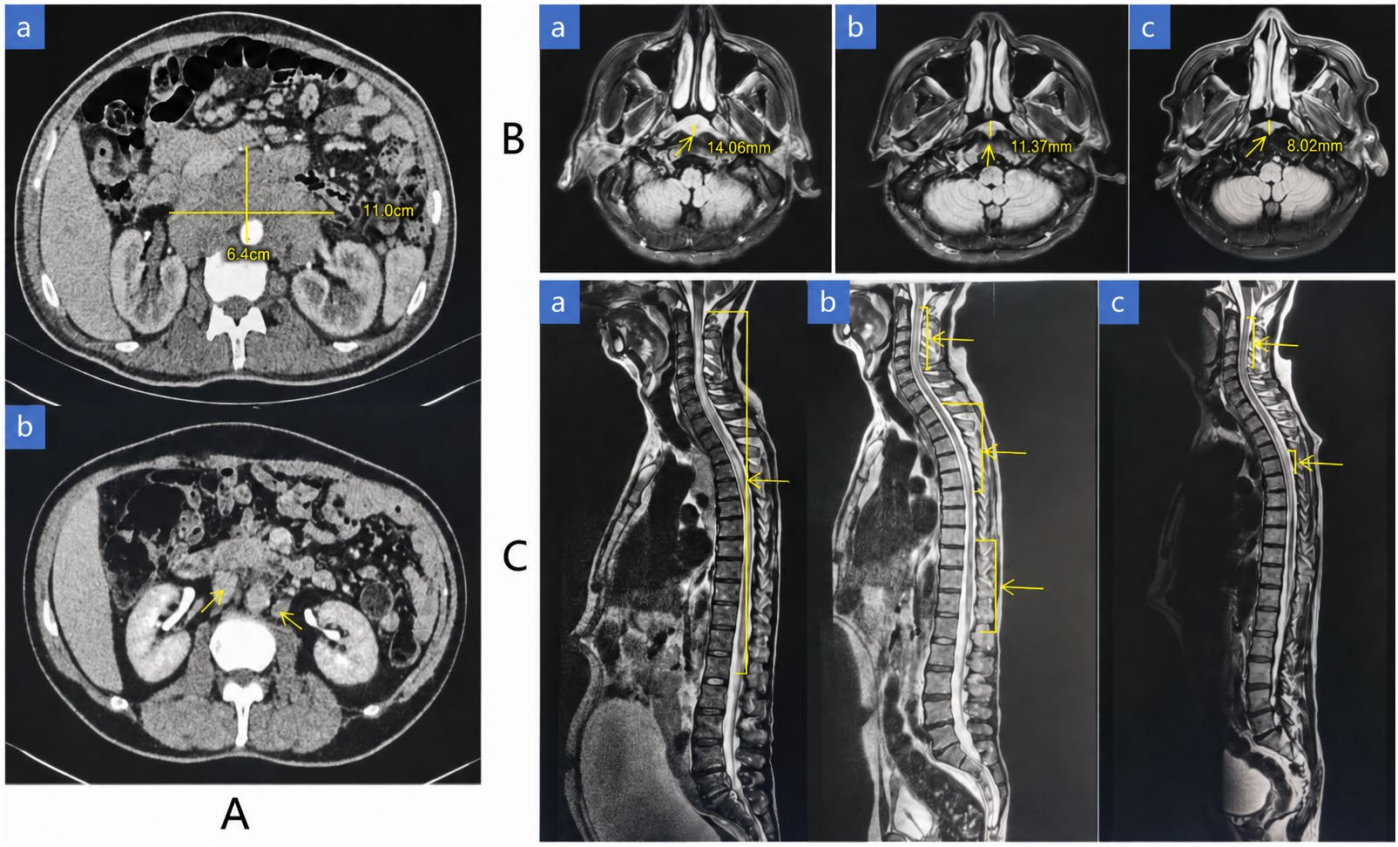
Imaging comparison at baseline and during treatment. (A) Contrast‐enhanced CT demonstrates reduction of the largest abdominal nodal mass from 11.0 × 6.4 cm at baseline (a) to 1.3 × 0.9 cm at Month 4 (b). The measurement calipers delineate the baseline mass in A(a), and the yellow arrows indicate residual abdominal nodal lesions in A(b). (B) Nasopharyngeal MR images show a reduction in soft‐tissue thickness from 14.06 mm at baseline (a) to 11.37 mm at Month 1 (b) and 8.02 mm at Month 4 (c). Yellow arrows indicate the nasopharyngeal soft‐tissue thickening. (C) Whole‐spine MR images demonstrate progressive improvement in swelling and signal abnormalities involving the lower medulla oblongata and spinal cord from baseline (a) to Month 1 (b) and Month 4 (c). Yellow brackets delineate the approximate craniocaudal extent of the abnormal spinal‐cord segments, while yellow arrows point to the corresponding abnormalities, showing extensive continuous involvement at baseline (a), three discontinuous residual segments at Month 1 (b), and two residual segments at Month 4 (c).

The patient was diagnosed with CLL/SLL with CNS involvement, Ann Arbor Stage IV, and bulky disease. The CLL‐IPI score was 4, corresponding to a high‐risk group.

### 2.1 Treatment and Response

Because paraparesis and urinary retention were rapidly worsening, an indwelling urinary catheter was inserted. Intravenous dexamethasone was administered at 10 mg once daily for 6 days in April 2023 as short‐course pretreatment and was then discontinued without tapering; no subsequent systemic dexamethasone course was documented. Intrathecal chemotherapy with methotrexate, cytarabine, and dexamethasone was given after lumbar puncture. Hydration, urine alkalinization, and diuresis were used during the initiation of systemic therapy.

Zanubrutinib was initiated in April 2023 at 160 mg twice daily. Venetoclax was started later in April 2023 at 20 mg once daily and was subsequently escalated weekly to 400 mg once daily. Baseline HBV serology showed HBsAg positivity, HBeAg negativity, and anti‐HBe and anti‐HBc positivity; HBV DNA was < 1.00 × 10^2^ IU/mL. Tenofovir amibufenamide 25 mg once daily was continued throughout antineoplastic treatment, and subsequent HBV DNA testing became undetectable. No additional antibacterial, antifungal, or *Pneumocystis jirovecii* pneumonia prophylaxis was documented in the retrievable records. A formal tumor‐lysis syndrome (TLS) risk classification and the detailed laboratory‐monitoring schedule during venetoclax dose escalation were not available in the retrievable contemporaneous medical records.

Night sweats, lower‐limb weakness, sensory symptoms, and urinary difficulty gradually improved. By 2 months, the urinary catheter had been removed and the patient could void independently with assistance. By 3 months, he could walk independently. At 4 months, bone‐marrow biopsy, bone‐marrow cytology and flow cytometry, and CSF cytology and flow cytometry were reported negative. CT showed marked regression of lymphadenopathy, with the largest residual lesion measuring approximately 13 × 9 mm. Nasopharyngeal soft‐tissue thickness decreased from 14.06 mm at baseline to 11.37 mm at Month 1 and 8.02 mm at Month 4. Whole‐spine MRI also demonstrated reduced spinal‐cord swelling and signal abnormality (Figure 1A(b),B(b,c),C(b‐c)).

According to the 2018 iwCLL recommendations, the Month‐4 response was assessed as complete remission [17]. The December 2024 PET/CT Deauville score of 2 was consistent with complete metabolic response under the Lugano classification [18].

### 2.2 Long‐Term Follow‐Up and Safety

Subsequently, the patient remained on oral venetoclax and zanubrutinib and received intrathecal triple chemotherapy (methotrexate/dexamethasone/cytarabine) via lumbar puncture every 6 months. Low‐level CSF measurable residual disease (MRD), assessed by flow cytometry, was detected in June 2024: 57 phenotypically abnormal monoclonal mature small B cells, representing 2.22% of nucleated cells; bone‐marrow MRD was negative. In November 2024, lumbar puncture and intrathecal treatment were performed, after which CSF MRD was negative. PET/CT in December 2024 showed substantial reduction in previously involved nodal sites with a Deauville score of 2, decreased spinal‐cord metabolic activity, and no residual PET/CT abnormality corresponding to the previous nasopharyngeal thickening or neurogenic bladder. Venetoclax was discontinued in April 2025 after approximately 2 years of combination therapy, and zanubrutinib monotherapy was continued.

During the September 2025 reassessment, bone‐marrow biopsy showed preserved trilineage hematopoiesis and no definite tumor involvement. Ultrasonography showed only small cervical, axillary, supraclavicular, and inguinal lymph nodes; no retroperitoneal mass was identified, and the spleen was not enlarged. Mild‐to‐moderate fatty liver, hepatic cysts, and gallstones were documented, without an established relationship to treatment.

At the latest documented hospitalization in April 2026, the patient was in generally stable condition, with normal bowel and bladder function. Bone‐marrow and CSF examinations were negative. Contrast‐enhanced brain MRI and whole‐spine MRI showed no evidence of intracranial or intraspinal tumor involvement; other findings included stable small lacunar infarcts, degenerative spinal changes, a probable T9 Schmorl node, and mild nasopharyngeal soft‐tissue thickening. Oral zanubrutinib was therefore stopped in April 2026. Blood counts remained generally stable throughout treatment; HBV DNA became undetectable during continued tenofovir amibufenamide therapy, and no HBV reactivation, tumor‐lysis syndrome, serious infection, treatment interruption, or other clinically significant toxicity was documented. The patient's clinical course, including the key diagnostic assessments, treatments, and outcomes, is summarized in Table 1.

**TABLE 1:** Clinical timeline of diagnosis, treatment, and response.

Time point	Assessment or treatment	Outcome
March–April 2022	PET/CT for longstanding lymphadenopathy; right axillary lymph‐node biopsy confirmed CLL/SLL.	Observed without treatment.
October 2022–March 2023	Night sweats and fatigue progressed; lower‐limb weakness and sensory symptoms developed.	Symptomatic systemic and neurologic progression.
April 2023	Whole‐spine MRI, PET/CT, marrow and CSF studies.	CNS‐involved Stage IV bulky CLL/SLL diagnosed.
April 2023	Dexamethasone plus intrathecal chemotherapy.	Urgent CNS‐directed treatment.
April 2023	Zanubrutinib 160 mg twice daily started.	Systemic targeted therapy.
April 2023	Venetoclax 20 mg daily started and escalated weekly to 400 mg daily.	Dual BTK/BCL‐2 inhibition.
Months 2–4	Urinary catheter removed; independent ambulation restored; marrow/CSF negative at Month 4; major CT/MRI improvement.	Deep clinical, marrow, CSF, and radiographic response.
June 2024	Bone‐marrow MRD negative; low‐level CSF MRD detected by flow cytometry (57 abnormal cells; 2.22% of nucleated cells).	Low‐level CNS disease detected.
November 2024	Lumbar puncture and intrathecal treatment.	CSF MRD negative.
December 2024	PET/CT Deauville score 2; reduced spinal‐cord metabolism.	Complete metabolic response.
April 2025	Venetoclax discontinued after approximately 2 years of combination therapy; zanubrutinib continued as monotherapy.	Continued systemic therapy with zanubrutinib alone.
September 2025	Marrow without definite tumor involvement; only small residual nodes on ultrasound.	Continued systemic disease control.
April 2026	Final documented clinical review; zanubrutinib discontinued.	Stable general condition; normal bowel and bladder function; marrow and CSF negative; brain and whole‐spine MRI showed no tumor involvement.

## 3 Discussion

This case illustrates several recurring challenges in CNS involvement CLL/SLL. Neurologic symptoms are nonspecific and require consideration of infection, autoimmune or inflammatory disease, Richter transformation, other malignancy, and unrelated neurologic disorders [1, 2, 3]. CSF flow cytometry and clonality testing increase diagnostic sensitivity but must be interpreted together with cytology, imaging, and the clinical syndrome because circulating CLL/SLL cells may contaminate CSF specimens [1, 2, 3, 7, 8]. In the present case, the high proportion of clonal B cells in CSF, IgH/IgK rearrangements, severe neurologic deficits, and concordant spinal‐cord and medullary abnormalities supported true CNS involvement. Additional records documented systemic CMV/EBV testing, HBV studies, antiglobulin tests, a thyroid profile, and high‐sensitivity C‐reactive protein, as reported above. However, detectable plasma and cell‐associated EBV DNA do not establish CNS infection, and no systematic CSF infectious panel or neuronal autoantibody panel was identified. Infectious or autoimmune CNS alternatives therefore cannot be completely excluded retrospectively.

The rapid initial improvement followed corticosteroid and intrathecal therapy as well as initiation of the oral doublet. Dexamethasone may reduce edema and produce direct lympholysis, while intrathecal methotrexate and cytarabine can rapidly reduce leptomeningeal disease. Accordingly, these interventions probably contributed materially to early neurologic recovery. The later reappearance of low‐level CSF MRD and its clearance after additional intrathecal therapy further supports the clinical relevance of the CNS‐directed component. This sequence precludes attribution of the outcome to zanubrutinib plus venetoclax alone.

Nevertheless, the systemic targeted regimen has a credible mechanistic and clinical rationale. BTK inhibition disrupts B‐cell receptor and microenvironmental survival signaling, can reduce compensatory prosurvival support from proteins such as MCL‐1 and BCL‐XL, and increases mitochondrial dependence on BCL‐2, thereby enhancing sensitivity to venetoclax [10]. Zanubrutinib has demonstrated systemic efficacy in both untreated and relapsed/refractory CLL/SLL [11, 12], while zanubrutinib‐containing venetoclax regimens and the direct zanubrutinib–venetoclax doublet have produced deep responses in systemic disease [13, 14]. Fixed‐duration ibrutinib–venetoclax studies provide additional class‐level support for dual BTK and BCL‐2 inhibition [15, 16].

For CNS disease, the evidence base remains far smaller. Responses to ibrutinib have been reported in CNS‐involved CLL/SLL [4, 7, 8], and venetoclax has been measured in CSF and incorporated into successful multimodal strategies [5, 6]. A 13‐case CNS lymphoma series demonstrated measurable CSF concentrations of zanubrutinib, providing pharmacokinetic support for CNS exposure but not establishing efficacy in CNS‐involved CLL/SLL [9]. Zanubrutinib was selected because it was already approved and clinically available in China for CLL/SLL, while Phase 3 data supported its activity in previously untreated CLL/SLL [11, 12]. As a more selective second‐generation BTK inhibitor, zanubrutinib had shown a more favorable cardiac safety profile than ibrutinib in a head‐to‐head CLL/SLL study, particularly a lower incidence of atrial fibrillation or flutter [11]. This favored zanubrutinib when prolonged BTK inhibition was anticipated, although the choice was not based on evidence of superior CNS efficacy or penetration. An HD‐MTX‐based regimen was considered less suitable because the patient had severe neurologic impairment, an ECOG performance status of 4, and urinary retention requiring catheterization. HD‐MTX administration requires intensive hydration, urine alkalinization, reliable monitoring of urine output and renal function, serial methotrexate‐level assessment, and leucovorin rescue; the patient's urinary dysfunction and poor functional status would have complicated this supportive‐care pathway. Moreover, evidence for HD‐MTX in CNS‐involved indolent CLL/SLL remains sparse and largely case‐based, and sensitivity comparable to that of primary CNS lymphoma or other aggressive CNS lymphomas cannot be assumed [6]. We therefore selected an oral systemic regimen directed against the underlying CLL/SLL, together with short‐course dexamethasone pretreatment and intrathecal therapy for urgent CNS‐directed control.

The clinically informative feature is the combination of rapid functional recovery with prolonged systemic control documented through serial marrow, CSF, PET/CT, and ultrasound assessments. The Month‐4 negative CSF result was followed by low‐level positivity in June 2024 and subsequent negativity after another intrathecal treatment. This pattern argues against describing the oral doublet as independently curative, while the approximately 2‐year course of zanubrutinib plus venetoclax, followed by zanubrutinib monotherapy after April 2025, may have contributed to sustained systemic control and complemented intermittent CNS‐directed therapy.

This report has important limitations. It describes a single patient; treatment components overlapped; CSF concentrations of zanubrutinib and venetoclax were not measured; and several historical clinical details could not be reliably retrieved, including a systematic CSF infectious or neuronal autoimmune workup, formal TLS risk classification, detailed venetoclax ramp‐up laboratory monitoring, and whether antibacterial, antifungal, or *Pneumocystis jirovecii* pneumonia prophylaxis was used. The treatment‐selection rationale was clarified retrospectively with the treating team rather than from a contemporaneous written comparative assessment. The absence of clinically significant toxicity documented in this individual cannot establish the general safety of the regimen. These limitations require cautious interpretation and prospective validation.

## 4 Conclusion

In this patient with CNS‐involved SLL and severe neurologic impairment, urgent dexamethasone and intrathecal chemotherapy followed by zanubrutinib plus venetoclax was associated with major neurologic recovery, complete remission at Month 4, and prolonged disease control. Low‐level CSF MRD later required additional intrathecal treatment. The oral regimen was administered without clinically significant toxicity in this patient. The case supports further investigation of zanubrutinib plus venetoclax as the systemic component of a multimodal, chemotherapy‐sparing strategy, but it cannot establish the independent efficacy, general safety, or standard‐of‐care status of the oral doublet.

## Author Contributions

**Wei Xie:** conceptualization, data curation, investigation, writing – original draft, writing – review and editing, methodology, formal analysis, validation. **Yanxue Yin:** data curation, investigation. **Jing Wang:** visualization, resources. **Guohui Cui:** conceptualization, project administration. **Lu Zhang:** conceptualization, supervision, writing – review and editing, project administration, funding acquisition.

## Funding

The authors have nothing to report.

## Ethics Statement

This study was conducted in accordance with the Declaration of Helsinki. Due to the retrospective, non‐interventional nature of this study, and since the patient data were fully anonymized prior to analysis, formal institutional review board (IRB) approval was waived by the Medical Ethics Committee of Union Hospital, Tongji Medical College, Huazhong University of Science and Technology.

## Consent

Written informed consent for publication of this case report and accompanying images was obtained from the patient.

## Conflicts of Interest

The authors declare no conflicts of interest.

## Data Availability

The data that support the findings of this study are available on request from the corresponding author. The data are not publicly available due to privacy or ethical restrictions.

## References

[1] P. Strati , J. H. Uhm , T. J. Kaufmann , et al., “Prevalence and Characteristics of Central Nervous System Involvement by Chronic Lymphocytic Leukemia,” Haematologica 101, no. 4 (2016): 458–465, 10.3324/haematol.2015.136556. PMC5004406 26819053

[2] A. Wanquet , R. Birsen , C. Bonnet , et al., “Management of Central Nervous System Involvement in Chronic Lymphocytic Leukaemia: A Retrospective Cohort of 30 Patients,” British Journal of Haematology 176, no. 1 (2017): 37–49, 10.1111/bjh.14387. 27858991

[3] L. Y. Qin , Y. Geng , J. F. Mu , et al., “Central Nervous System Involvement in Chronic Lymphocytic Leukemia: A Case Report and Review of Literature,” Journal of Medical Case Reports 18 (2024): 315, 10.1186/s13256-024-04639-4. PMC11234772 38982482

[4] A. Wanquet , R. Birsen , R. Lemal , M. Hunault , V. Leblond , and T. Aurran‐Schleinitz , “Ibrutinib Responsive Central Nervous System Involvement in Chronic Lymphocytic Leukemia,” Blood 127, no. 19 (2016): 2356–2358, 10.1182/blood-2016-02-697193. 26994148

[5] G. Reda , R. Cassin , G. Dovrtelova , et al., “Venetoclax Penetrates in Cerebrospinal Fluid and May Be Effective in Chronic Lymphocytic Leukemia With Central Nervous System Involvement,” Haematologica 104, no. 5 (2019): e222–e223, 10.3324/haematol.2018.213157. PMC6518888 30765472

[6] G. Beziat , M. Gauthier , C. Protin , et al., “Venetoclax With High‐Dose Methotrexate and Rituximab Seem Effective and Well‐Tolerated in the Treatment of Central Nervous System Involvement of Chronic Lymphocytic Leukemia: A Case Report,” Clinical Case Reports 8, no. 2 (2020): 269–273, 10.1002/ccr3.2580. PMC7044374 32128171

[7] A. V. Naydenov and L. P. Taylor , “Leptomeningeal Carcinomatosis in Chronic Lymphocytic Leukemia: A Case Report and Review of the Literature,” Oncologist 24, no. 9 (2019): 1237–1245, 10.1634/theoncologist.2018-0619. PMC6738317 30842245

[8] T. Nakanishi , T. Ito , S. Fujita , et al., “Refractory Chronic Lymphocytic Leukemia With Central Nervous System Involvement: A Case Report With Literature Review,” Journal of Blood Medicine 11 (2020): 487–502, 10.2147/JBM.S271335. PMC7767717 33380856

[9] Y. Zhang , Y. Li , Z. Zhuang , et al., “Preliminary Evaluation of Zanubrutinib‐Containing Regimens in DLBCL and the Cerebrospinal Fluid Distribution of Zanubrutinib: A 13‐Case Series,” Frontiers in Oncology 11 (2021): 760405, 10.3389/fonc.2021.760405. PMC8739956 35004280

[10] J. Deng , E. Isik , S. M. Fernandes , J. R. Brown , A. Letai , and M. S. Davids , “Bruton's Tyrosine Kinase Inhibition Increases BCL‐2 Dependence and Enhances Sensitivity to Venetoclax in Chronic Lymphocytic Leukemia,” Leukemia 31, no. 10 (2017): 2075–2084, 10.1038/leu.2017.32. PMC5555835 28111464

[11] J. R. Brown , B. Eichhorst , P. Hillmen , et al., “Zanubrutinib or Ibrutinib in Relapsed or Refractory Chronic Lymphocytic Leukemia,” New England Journal of Medicine 388, no. 4 (2023): 319–332, 10.1056/NEJMoa2211582. 36511784

[12] C. S. Tam , J. R. Brown , B. S. Kahl , et al., “Zanubrutinib Versus Bendamustine and Rituximab in Untreated Chronic Lymphocytic Leukaemia and Small Lymphocytic Lymphoma (SEQUOIA): A Randomised, Controlled, Phase 3 Trial,” Lancet Oncology 23, no. 8 (2022): 1031–1043, 10.1016/S1470-2045(22)00293-5. 35810754

[13] J. D. Soumerai , A. R. Mato , A. Dogan , et al., “Zanubrutinib, Obinutuzumab, and Venetoclax With Minimal Residual Disease‐Driven Discontinuation in Previously Untreated Patients With Chronic Lymphocytic Leukaemia or Small Lymphocytic Lymphoma: A Multicentre, Single‐Arm, Phase 2 Trial,” Lancet. Haematology 8, no. 12 (2021): e879–e890, 10.1016/S2352-3026(21)00307-0. PMC9326222 34826411

[14] M. Shadman , T. Munir , S. Ma , et al., “Zanubrutinib and Venetoclax for Patients With Treatment‐Naïve Chronic Lymphocytic Leukemia/Small Lymphocytic Lymphoma With and Without del(17p)/TP53 Mutation: SEQUOIA Arm D Results,” Journal of Clinical Oncology 43, no. 21 (2025): 2409–2417, 10.1200/JCO-25-00758. 40448577

[15] C. S. Tam , J. N. Allan , T. Siddiqi , et al., “Fixed‐Duration Ibrutinib Plus Venetoclax for First‐Line Treatment of CLL: Primary Analysis of the CAPTIVATE FD Cohort,” Blood 139, no. 22 (2022): 3278–3289, 10.1182/blood.2021014488. PMC11022982 35196370

[16] T. Munir , D. A. Cairns , A. Bloor , et al., “Chronic Lymphocytic Leukemia Therapy Guided by Measurable Residual Disease,” New England Journal of Medicine 390, no. 4 (2024): 326–337, 10.1056/NEJMoa2310063. 38078508

[17] M. Hallek , B. D. Cheson , D. Catovsky , et al., “iwCLL Guidelines for Diagnosis, Indications for Treatment, Response Assessment, and Supportive Management of CLL,” Blood 131, no. 25 (2018): 2745–2760, 10.1182/blood-2017-09-806398. 29540348

[18] B. D. Cheson , R. I. Fisher , S. F. Barrington , et al., “Recommendations for Initial Evaluation, Staging, and Response Assessment of Hodgkin and Non‐Hodgkin Lymphoma: The Lugano Classification,” Journal of Clinical Oncology 32, no. 27 (2014): 3059–3068, 10.1200/JCO.2013.54.8800. PMC4979083 25113753

